# 2025 American Thyroid Association Management Guidelines for Adult Patients With Differentiated Thyroid Cancer: What's New for Surgeons?

**DOI:** 10.1002/ohn.70367

**Published:** 2026-08-06

**Authors:** Chau Nguyen, Amanda Silver Karcioglu, Joseph Scharpf

**Affiliations:** ^1^ Ventura County Medical Center Ventura California USA; ^2^ Endeavor Health–teaching affiliate of the University of Chicago Evanston Illinois USA; ^3^ Cleveland Clinic Cleveland Ohio USA

**Keywords:** ATA guidelines, new, surgeons, thyroid cancer

## Abstract

The American Thyroid Association (ATA) has published an update to the 2015 management guidelines for adult patients with thyroid nodules and differentiated thyroid cancer (DTC). This updated version focuses solely on the management of differentiated thyroid cancer, offering a comprehensive set of guidelines for all stakeholders in the care of differentiated thyroid cancer, but no longer addresses the management of thyroid nodules in adults. Herein, we highlight the key updates and differences between the two versions, emphasizing those most pertinent to endocrine neck surgeons. These guidelines will form the basis for differentiated thyroid cancer care over the next few years, affecting millions of patients. For maximal impact, they will need to be widely disseminated, discussed, and dissected, to ultimately result in practice changes befitting the latest evidence‐based care.

The expert panel remains largely unchanged. A third of the authors are new including for the first time a patient advocate and an expert in systematic reviews/meta‐analyses/guidelines.

Grading of recommendations is reported as strong or conditional (replacing the term “weak”). Good Practice Statements are now provided where benefit is believed to be high despite lack of evidence; for example, in cases where evidentiary collection would not be feasible. These statements required unanimous consensus.^1^, ^2^ Also new is the DATA framework developed for Diagnosis, risk/benefit Assessment, Treatment decisions, and response Assessment. This framework is intended to guide clinicians in the management of initial or recurrent/persistent differentiated thyroid cancer.

## Highlight of Key and New Recommendations

The 2015 goals of initial therapy have been streamlined and now include consideration of multimodal treatment options to decrease persistence, recurrence, and metastasis while balancing the risks of morbidity of treatment.

Guidance for the management of non‐invasive follicular thyroid neoplasm with papillary‐like nuclear features (NIFTP), which comprise 2.1%–9.6% of all follicular thyroid neoplasms, is new as this entity was first described in 2016. Diagnosis is made on pathological examination, and further treatment, such as completion thyroidectomy, is not advised due to the very low malignant potential, a hallmark feature. The necessity of postoperative monitoring, however, remains unclear. This is the first recommendation of the guidelines:

RECOMMENDATION 1

NIFTP and other tumors of uncertain malignant potential (Follicular Tumor of Uncertain Malignant Potential and Hyalinizing Trabecular Tumor) are diagnosed pathologically and have a very low malignant potential (lower than the lowest‐risk DTC). Further treatment with completion thyroidectomy/lymphadenectomy and/or RAI is not advised. The need for, and frequency of, postoperative monitoring of these tumors is uncertain. **(Good Practice Statement).**

Recommendations for genetic testing, including germline testing in syndromic cases such as Cowden/PTEN hamartoma tumor syndrome or children with non‐medullary thyroid cancer, are delineated. Separately, for non‐syndromic patients with familial non‐medullary thyroid cancer, a lack of evidence supporting the usefulness of genetic testing is cited. Both are conditional recommendations.

The role for high volume thyroid surgeons (defined as greater than 25‐50 thyroidectomies/year) is elucidated, especially for complex cases requiring more extensive surgery. A strong recommendation with high certainty evidence is given for patients with thyroid cancer to be referred to a high‐volume surgeon, based on evidence of improved outcomes and lower complication rates. The guidelines authors find “[w]hen hospital volume and surgeon volume are both considered, on average, high‐volume surgeons are associated with lower complication rates, lower hospital mortality, and lower cost, whereas high‐volume centers are associated primarily with lower cost and shorter lengths of stay.” Their research into the exact number of total thyroidectomy annual case volume needed yielded greater than 25 to set the floor of this range. They do note the challenges clinicians and patients face given the reality of a relatively small number of high‐volume surgeons who are more likely to be in urban centers. It is acknowledged that population‐level data isn't always applicable to individual surgeon and patient circumstances, and there is an important role for patient preference.

For small papillary thyroid cancers (≤1 cm), comprising 39% of new thyroid cancer cases in 2008‐9 and whose diagnosis contributed to the increased incidence of thyroid cancer up to 2013, active surveillance is now codified in the guidelines as a conditional recommendation. Surveillance ultrasound is recommended, whereas measurement of serum thyroglobulin and anti‐thyroglobulin antibodies is not. There is less clarity, however, on when to proceed to surgery. A good practice statement suggests that biopsy‐proven lymph node metastases, increase in size ≥3 mm on ultrasound, distant metastases, posterior growth, and patient factors are all considerations to pursue operative intervention. Active surveillance is not recommended for tumors with aggressive histology or behavior such as extrathyroidal extension, recurrent nerve invasion, or metastatic disease. It is noted that studies of active surveillance have excluded tumors around the posterior gland or trachea, due to concern of invasion into critical nearby structures such as the airway or recurrent nerve, should the tumor progress. Alternatively, percutaneous ablation involving radiofrequency (RFA), microwave, laser, or ethanol ablation are newer techniques which may be offered to select patients with cT1a disease.

Continuing the trend toward more conservative surgical treatment in differentiated thyroid cancer, for unilateral low‐risk T2N0M0 thyroid cancers (2–4 cm) diagnosed by cytology or molecular testing, Recommendation 15 states, “thyroid lobectomy may be the **preferred** initial surgical treatment due to significantly lower risk and side effects. However, the patient and treatment team may adopt total thyroidectomy to enable RAI [radioactive iodine] administration and/or enhance follow‐up based on disease features, suspicious contralateral nodularity, and/or patient preferences. When thyroid lobectomy is offered as initial treatment, the patient should be counseled about the possibility of conversion to total thyroidectomy or need for subsequent completion thyroidectomy if higher‐risk factors emerge intraoperatively or postoperatively.” In comparison, the 2015 equivalent conditional recommendation stated: “For patients with thyroid cancer >1 and <4 cm without extrathyroidal extension, and without clinical evidence of any lymph node metastases (cN0), the initial surgical procedure can be **either** a bilateral procedure (near‐total or total thyroidectomy) or a unilateral procedure (lobectomy). Thyroid lobectomy alone may be sufficient initial treatment for low‐risk papillary and follicular carcinomas; however, the treatment team may choose total thyroidectomy to enable RAI therapy or to enhance follow up based upon disease features and/or patient preferences.” [Emphasis ours] Despite equivocal data with “half [of studies reviewed] showing no difference in recurrence or survival but with higher complication rates for total thyroidectomy [while the] other half demonstrate statistically significant, lower recurrence rates with total thyroidectomy compared with lobectomy alone”, the current guidance for lobectomy reflects the panel's emphasis on the significantly lower rate of complications, better quality of life, and limited oncologic benefit of a more aggressive surgical approach.

Similarly, while the overall recommendation for prophylactic central compartment neck dissection has not changed and “may be considered in patients with PTC [papillary thyroid cancer] and clinically uninvolved lymph nodes (cN0) who have advanced primary tumors (T3 or T4) or for whom the information will be used to plan further steps in therapy,” there is now a strong recommendation *against* this practice for most low risk cT1‐2 cancers.

Management of thyroglossal duct carcinoma is discussed for the first time. A Sistrunk procedure is recommended as the initial surgery, with the possibility of thyroidectomy in the presence of significant/suspicious nodules, and/or more advanced disease such as nodal metastases or invasion into local structures.

Recommendations for intraoperative neural monitoring (IONM) have been expanded. While visual identification of the recurrent laryngeal nerves (RLN) remains paramount (Good Practice Statement), use of monitoring is further specified to reduce injury rates during total thyroidectomy for malignancy or reoperative surgery. Similarly, in cases of malignancy, the external branch of the superior laryngeal nerve (EBSLN) may be identified and monitored to improve voice outcomes. A novel Good Practice Statement involves intraoperative vagal nerve or proximal RLN stimulation to assess the function and integrity of the nerve prior to removal of the contralateral lobe. If there is identified nerve injury, referral to speech language pathology and a physician voice specialist is suggested.

Prior guidelines for parathyroid preservation were general, recommending identification and preservation. There is now guidance for auto‐transplantation in case of gland devascularization or inadvertent removal, following frozen section confirmation of benign parathyroid tissue prior to re‐implantation. A strong recommendation is given to use parathyroid hormone‐directed calcium and vitamin D supplementation to decrease postoperative hypocalcemia following bilateral surgery, central neck dissection, or completion surgery.

Use of a drain is specifically discouraged in “most circumstances” due to increased length of stay, potential for infection, and lack of efficacy in reducing hematoma development.

A significant change of the updated 2025 guidelines involves assessment and risk of postoperative recurrence using a new ATA Risk stratification system (RSS), which was developed to be used with the 8^th^ edition American Joint Committee on Cancer (AJCC) staging system. There are now 3 separate tracks (for papillary, follicular, and oncocytic thyroid cancer) with four risk categories. The intermediate risk category is further sub‐divided now into a low‐intermediate and intermediate‐high. Overall, this change appears to simplify access to the necessary information. Application of this assessment is recommended within 90 days of surgery. Molecular profiling of the surgical specimen is not required. Table 1 summarizes some key changes between versions.

**Table 1:** Risk Stratification Structure 2015 Versus 2025 ATA Guidelines

Feature	2015 ATA guidelines	2025 ATA guidelines
System Structure	A single three‐tiered categorical system for all DTC (Low, Intermediate, High).	Three separate tracks tailored to Papillary (PTC), Follicular (FTC/IEFVPTC), and Oncocytic (OTC) cancers.
Risk Categories	Three levels: Low, Intermediate, and High.	Four levels: Low, Low‐Intermediate, Intermediate‐High, and High.
Recurrence Risk Percentages	A continuum from ~ Low: <1–5% Intermediate: 5–20% High: >20%	Low: <10% Low‐Intermediate: 10–15% Intermediate‐High: 16–30% High: >30%.
Oncocytic (Hürthle Cell) Cancer	Classified as a subtype of Follicular Thyroid Cancer.	Recognized as a distinct third form of DTC with a dedicated risk track.
Invasive Encapsulated Follicular Variant (IEFVPTC)	Grouped generally with other PTC variants.	Grouped with Follicular Thyroid Cancer due to similar clinical behavior and vascular invasion patterns.
NIFTP	Not included in 2015 risk stratification (entity described in 2016).	Formally defined as a very low‐risk neoplasm with extremely low malignant potential.
AJCC Integration	Used in conjunction with AJCC 7th edition staging.	Designed to be used with AJCC 8th edition staging, which emphasizes disease‐specific survival.
Lymph Node Assessment	Low risk included N0 or ≤5 micrometastases (< 0.2 cm).	Risk continues to use number > 5 as intermediate risk and size threshold of > 3 cm for high intermediate; central neck recurrence threshold potentially increased from >8 mm to ≥15 mm.
Extrathyroidal Extension (ETE)	Microscopic ETE was used to define Intermediate risk (and T3 staging).	Microscopic ETE removed from AJCC 8th T3 staging; only gross ETE (strap muscles or major structures) significantly impacts risk.
Vascular Invasion	FTC with <4 vessels was low risk; ≥4 vessels was high risk.	Categorization maintained but more strictly defined as tumor cells invading through a vessel wall covered with endothelium.

*Note*: The publisher for this copyrighted material is Mary Ann Liebert, Inc. publishers.

Assessment of the clinical response to surgery continues to be defined by the ATA Response Criteria: excellent response, indeterminate response, biochemical incomplete response, and structural incomplete response. A strategy for application of the DATA framework in a dynamic ongoing assessment would be: 1. Diagnosis and Assessment using the ATA RSS following surgery, 2. defining the Treatment response over time, and 3. continual Assessment of risk of recurrence during follow‐up surveillance.

Noteworthy to the 2025 guidelines is a role for the de‐escalation and in select cases discontinuation of monitoring low‐risk DTC (Recommendation 48). Patients who have undergone a total thyroidectomy with or without RAI with a sustained excellent response may be considered for cessation of US monitoring after 5 to 8 years and biochemical monitoring after 10‐15 years. An excellent biochemical response is defined as thyroglobulin (Tg) < 2.5 ng/mL with undetectable thyroglobulin antibody (TgAb) for patients post total thyroidectomy without RAI, and Tg < 0.2 post total thyroidectomy with RAI. For patients who have undergone a lobectomy, if the initial US is negative, subsequent surveillance US may be performed every 1 to 3 years for a period of 5 to 8 years after. Contralateral nodules should be monitored as recommended in the ATA guidelines, and measurement of Tg is not recommended, provided an initial postoperative Tg is not significantly elevated.

Recommendation 52 addresses the management of locoregional recurrent, residual, or progressive DTC. Re‐operation should be individualized considering the extent of prior surgery, disease size, location, and rate of growth, and patient factors. Conditional recommendations are given for percutaneous ethanol injection and radiofrequency ablation, as alternatives to surgery in patients deemed higher surgical risk or refusing surgery (both modalities were discussed in the prior guidelines without level of recommendation). In addition, current research points to a possible increased surgical threshold for management of central neck recurrence from >8 mm (in the smallest dimension) to >15 mm.

In the previous guidelines, management of thyroid nodules during pregnancy and approach to malignant or indeterminate cytology were discussed (Recommendations 30‐31). This section is updated in the newest guidelines to “Considerations managing pregnant patients with DTC” (Recommendation 81). A conditional recommendation is given to deferring surgery until after delivery for most patients, which remains similar to the prior weak recommendation to monitor cancerous nodules with ultrasound in early pregnancy. If surgery is required, then the second trimester is reaffirmed as the best time to operate. The clinical circumstances that would lead to this are again not clearly delineated, although growth (20% increase in nodule size) and malignant lymph nodes are mentioned in the 2015 paper as potential reasons.

The panel omitted recommendation 72 from the 2015 guidelines specifically endorsing the role of surgery when feasible for invasive aerodigestive tract disease, along with RAI and external beam radiation, which was rated as strong.

The last set of recommendations (Recommendations 82‐84 presented as Good Practice Statements) address cancer survivorship. Discussion of long‐term treatment side effects, provision of resources for dealing with financial toxicity, and support services to help patients cope with their cancer are addressed.

For ease of reference, the changes discussed herein are paraphrased in Tables 2 and 3.

**Table 2:** Key New Recommendations

*NIFTP (Non‐invasive follicular thyroid neoplasm with papillary‐like nuclear features)*
RECOMMENDATION 1—NIFTP NIFTP is a thyroid tumor with low potential for cancer transformation, and no additional treatment with completion surgery or RAI is recommended. Appropriate follow‐up of these patients is currently unclear.
*Genetic testing*
RECOMMENDATION 2*—*Whom should obtain germline genetic testing Several clinical scenarios may warrant testing, including cases of cribriform morular carcinoma (APC gene), Cowden/PTEN Harmartoma Tumor Syndrome, pediatric patients with NMTC, or suspicion of a hereditary condition in patient and family pedigree.
RECOMMENDATION 3*—*Genetic testing in patients with non‐syndromic FNMTC The evidence does not support routine testing in these patients.
RECOMMENDATION 4*—*Family member screening for FNMTC FNMTC is defined as families in which three or more first or second degree related individuals have NMTC. US screening may be offered for first degree relatives in these cases.
RECOMMENDATION 5*—*Germline genetic testing in patients with alterations in tumor sampling If genomic testing is done and reveals a cancer predilection, patients should be further evaluated and referred for genetic counseling. *Surgeon experience* ▪ RECOMMENDATION 6*—*Surgical experience and complications Significant evidence shows improved outcomes in thyroid cancer treatment for high‐volume thyroid surgeons (> 25 to 50 thyroidectomies annually). Referral to a high‐volume surgeon should therefore be considered, especially in patients with invasive or extensive disease, or older patients with a worse prognosis.
*Preoperative molecular testing*
RECOMMENDATION 10*—*Preoperative molecular testing and extent of surgery Routine preoperative molecular testing is not supported.
*Active Surveillance (AS)*
RECOMMENDATION 11*—*AS Patients with small early DTC T1a may be candidates for active surveillance. Alternatively, percutaneous ablation with radiofrequency, microwave, laser, or ethanol may be considered.
RECOMMENDATION 12*—*How should patients be surveilled Serial ultrasound should be used to monitor these patients.
RECOMMENDATION 13*—*Utility of Tg and TgAb in AS Routine serum Tg or TgAB should not be followed for patients in active surveillance.
RECOMMENDATION 14*—*When is surgery indicated in AS Locoregional or distant metastases, tumor increase >3 mm, extrathyroidal extension or posterior extension, and patient preference are all reasons to consider operative intervention.
*Thyroglossal Duct Cancer (TGDCa)*
RECOMMENDATION 17*—*Surgical management of TGDCa A Sistrunk procedure is adequate surgery except in cases of advanced disease, such as for gross invasion or metastatic disease. Thyroidectomy can be considered in these cases, along with large primary tumors or those in whom nodular thyroid disease requires treatment due to factors including size or specific characteristics.
RECOMMENDATION 18*—*Completion total thyroidectomy in TGDCa For patients with high‐risk features or metastatic disease, completion total thyroidectomy may be performed. Similar to the surgical approach detailed above, it may also be done for large primary disease, potential multicentric disease, nodular thyroid disease requiring treatment, or to enable RAI.
*Use of a Drain*
RECOMMENDATION 25*—*Postoperative use of drains Placement of a drain is not routinely recommended.
*De‐escalation or discontinuation of monitoring for low‐risk DTC*
RECOMMENDATION 48*—*De‐escalation of monitoring in low‐risk DTC Patients with low‐risk DTC treated with total thyroidectomy with or without RAI and an excellent response five to eight years following initial treatment may be followed with biochemical markers every one to two years thereafter without ultrasound exams. After 10‐15 years of consistent excellent response, these patients may be considered as being in complete remission and do not need further testing. In patients who have undergone lobectomy for low‐risk DTC, US monitoring for disease recurrence may be done every one to three years for 5‐8 years, provided the initial US exam is negative. Tg testing is not recommended routinely.
*Treatment for locoregional recurrent, residual or progressive disease*
RECOMMENDATION 52*—*Treatment for locoregional recurrent, residual, or progressive DTC Factors to consider in the decision to treat are rate of growth, size, location, and patient choice. Alternatives to surgical management are radiofrequency ablation or percutaneous ethanol injection.
*Cancer Survivorship*
RECOMMENDATION 82*—*Long‐term survivorship after thyroid cancer Patients should be informed of long‐term effects of treatment, along with plans for management and monitoring.
RECOMMENDATION 83*—*Financial hardships facing thyroid cancer patients Clinicians should be aware that some patients may experience financial hardships and be prepared to discuss issues and offer potential resources accordingly.
RECOMMENDATION 84*—*Psychosocial concerns in survivors Clinicians should be prepared to help patients navigate psychosocial concerns and avail them of support services as needed.

*Note*: The publisher for this copyrighted material is Mary Ann Liebert, Inc. publishers.

**Table 3:** Key Changes to Recommendations

Central Neck Dissection
RECOMMENDATION 19 It is not recommended for cT1 or T2 cN0 PTC and for FTC.
*Recurrent laryngeal nerve assessment*
RECOMMENDATION 23‐ Following initial lobectomy, intraoperative confirmation of nerve function should be assessed by proximal RLN or vagal nerve stimulation.
*Parathyroid gland management*
RECOMMENDATION 24 In cases of inadvertent parathyroid removal or devascularization, auto‐transplantation should be performed into adjacent muscle tissue after confirmation of parathyroid origin.
*Risk of recurrence and initial assessment*
RECOMMENDATION 28 The ATA Risk Stratification System is recommended to assess patients within three months of operation. Evaluating pathology specimens with molecular profiles is not recommended. Thyroid cancer in pregnancy ▪ RECOMMENDATION 81 Most surgeries may safely wait until after delivery unless there is clinically significant progression, wherein surgery is preferably performed in the second trimester. Monitoring with US as clinically indicated is suggested.

*Note*: The publisher for this copyrighted material is Mary Ann Liebert, Inc. publishers.

## Summary

In the 10 years since the prior guidelines, significant progress continues in the management of thyroid cancer. The updated guidelines offer a comprehensive resource for the care of differentiated thyroid cancer, and the panel's herculean task is to be commended.

There remain, nevertheless, areas where greater clarity is hoped for in future versions. Although multifocal thyroid cancer disease is mentioned, the table 5 recommendation on multifocality could be made clearer, and specific guidance to its management in the form of a Recommendation would help. For instance, should multifocal disease in a lobectomy specimen prompt completion surgery?^3^, ^4^ According to the authors, lobectomy would be sufficient unless the multifocality was bilateral, but this is often found on final pathologic diagnosis only. And whereas the guidelines for recurrent/persistent disease are currently broad, with additional research, future recommendations may be refined. For EBSLN identification, electrophysiologic identification in the literature has been found to be superior,^5^ and although the updated guidelines reinforce the importance of identification and preservation of vital structures, IONM continues to be advocated mostly only for difficult cancer cases.

More than 10,000 papers referencing thyroid cancer have been published since the end date of the panel's literature review July 1, 2024, (Pubmed search “thyroid cancer” 7/1/24*—*03/27/26). It is our hope, therefore, that future guidelines may be updated in shorter interval as deemed necessary based on advances in the field. For that effort, an infusion of new experts would be welcomed to represent a greater diversity of clinical experiences and expertise.

## Author Contributions

**Chau Nguyen**, MD, substantial contributions to the conception or design of the work; or the acquisition, analysis, or interpretation of data for the work; drafting the work or revising it critically for important intellectual content; and final approval of the version to be published; AND 4) agreement to be accountable for all aspects of the work in ensuring that questions related to the accuracy or integrity of any part of the work are appropriately investigated and resolved. **Amanda Silver Karcioglu**, MD, drafting the work or revising it critically for important intellectual content; final approval of the version to be published; and agreement to be accountable for all aspects of the work in ensuring that questions related to the accuracy or integrity of any part of the work are appropriately investigated and resolved. **Joseph Scharpf**, MD, drafting the work or revising it critically for important intellectual content; final approval of the version to be published; and agreement to be accountable for all aspects of the work in ensuring that questions related to the accuracy or integrity of any part of the work are appropriately investigated and resolved.

## Disclosures

### Competing interests

Chau Nguyen, MD, Consultant, Neurovision Medical. The remaining authors declare no conflicts of interest.

### Funding

None.
